# MRSA panophthalmitis in a brittle diabetic

**DOI:** 10.1186/s12348-023-00344-3

**Published:** 2023-04-17

**Authors:** Erin Flynn, Stephen Lesche, Sabita Ittoop, Tamer Mansour, Stephanie Barak, Keith James Wroblewski

**Affiliations:** Department of Ophthalmology, George Washington University, 2150 Pennsylvania Avenue Suite 2A, Washington, DC, 20037 USA

## Introduction

Endophthalmitis is defined as infection of the inner coats or substance of the eye. This condition is caused through exogenous or endogenous sources that act as a nidus for infection. Exogenous cases of endophthalmitis typically occur from ocular trauma or as a postoperative complication. Endogenous causes of endophthalmitis are much less common, comprising only 5–10% of endophthalmitis cases [1]. Etiologies of endogenous endophthalmitis (EE) include systemic infections by fungi or bacteremia [2]. Common causative bacteria of EE in North America include Staphylococcus aureus, group B streptococci, and Streptococcus pneumoniae [3]. In Asian countries, the most common bacteria found in EE are gram negative organisms including Klebsiella spp, Escherichia coli, Pseudomonas aeruginosa, and Neisseria meningitidis [3]. However, only 0.04–0.05% of patients with bacteremia have EE as a complication; this rate increases to 0.2% if the bacteremia is Methicillin-resistant Staphylococcus aureus (MRSA) bacteremia [4]. Endogenous bacterial endophthalmitis (EBE) can present with a range of symptoms from asymptomatic to eye pain with conjunctival injection and reduced vision [5]. While monocular cases are most common, bilateral involvement of the eyes has been reported in 19–33% of cases [6]. Of patients who have been diagnosed with EE, many were also found to have comorbidities, including diabetes mellitus, renal failure, and immunocompromised status [2]. In this case, we present a patient with several risk factors who presented with a bilateral case of endogenous endophthalmitis from MRSA bacteremia. The patient passed during his care and his mother gave verbal and signed permission for this report.

## Case presentation

A 30-year-old male presented with bilateral eye swelling and MRSA bacteremia from an outside hospital. His past medical history included poorly controlled type 1 diabetes mellitus, end stage renal disease on dialysis, heart failure with reduced ejection fraction, and bilateral below the knee amputations. He initially presented to the outside hospital with malaise and was found to have MRSA bacteremia secondary to a permanent central line catheter infection. The patient had a history of several infected catheter infections and had a total of six replacements in a one year span. The patient was started on daptomycin and ceftaroline and cleared five series of blood cultures but developed bilateral orbital swelling.

Due to his uncontrolled diabetes, his visual acuity at baseline on presentation was light perception in the right eye and no light perception in the left eye. Upon GW admission, his visual acuity was bare LP OD and NLP OS. Initial blood cultures at the outside hospital were positive for Methicillin-resistant staph aureus (MRSA) but five subsequent blood cultures performed during the subsequent hospital admission to GW were negative. On initial examination, there was significant proptosis and periorbital edema as well as erythema and tenderness of the left eye. There was 360 degrees of chemosis, and the cornea showed early signs of band keratopathy and stromal edema. Anterior chamber and lens were difficult to evaluate due to the high degree of corneal decompensation. The right eye had less extensive erythema and tenderness without proptosis. There was no chemosis, and the cornea showed early band keratopathy with keratic precipitates on the endothelium. The anterior chamber was difficult to evaluate for cell. Iris was responsive to light although sluggish. Lens was clear. A B-scan was performed in both eyes. In the right eye, it showed a tractional retinal detachment involving the macula. In the left eye, B scan demonstrated a tented globe and a total funnel retinal detachment along with hyperechoic opacities in the vitreous which could represent vitreous cells, a fibrous membrane, or hemorrhage.

CT imaging showed an enlarged, elongated and proptotic left globe with scleral irregularity and discontinuity. An additional MRI revealed swelling that extended into the post-septal and intraconal space with increased density in the posterior compartment. These findings were consistent with endophthalmitis. The left eye wound prior to enucleation was positive for many polymorphonuclear leukocytes with gram positive cocci, later found to be MRSA.

With worsening bilateral periorbital swelling as well as concern for intracranial involvement/spread and for scleral melt on the left eye, the patient was taken for left eye enucleation with wound drainage/antibiotic irrigation. Enucleation and orbital debridement of the right eye was considered, but ultimately decided against. At the time of the surgery, a penetrating scleral wound approximately 11–12 mm was found extending posteriorly infero-nasal from the limbus to behind the insertion of the superior rectus. Purulent material was aspirated from within the globe. The sclera was found to be rigid and brittle, and the muscle insertions were unable to be isolated or preserved due to fibrosis. Wound cultures and fluid samples were taken and positive for MRSA. It was presumed that the endophthalmitis resulted in an orbital cellulitis from ‘spill-over.’

The patient alternated between systemic vancomycin and daptomycin given concern for intermediate resistance to vancomycin throughout his hospital stay. The patient continued to receive Moxifloxacin in the right eye and Tobramycin-Dexamethasone ointment along the eyelid margin in the left eye. The patient passed after discharge from our hospital.

## Pathology

**Fig. 1:**
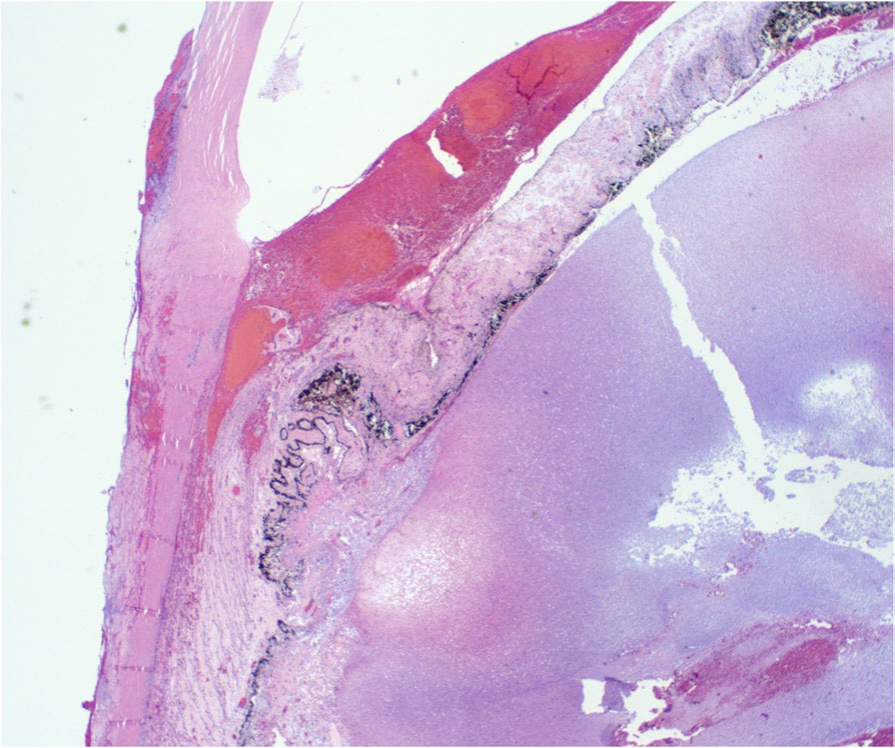
Shows a medium power view of the peripheral cornea peripheral iris and ciliary body. There is a thick hyphema layer blocking the angle and the iris is grossly thickened. There is dense inflammatory infiltrate focused just posterior to the ciliary body with florid necrosis

The entire globe was represented in the slide that was distributed and it shows extensive intra-ocular, scleral and episcleral acute inflammation with multifocal abscess formation. Figure 1.

**Fig. 2:**
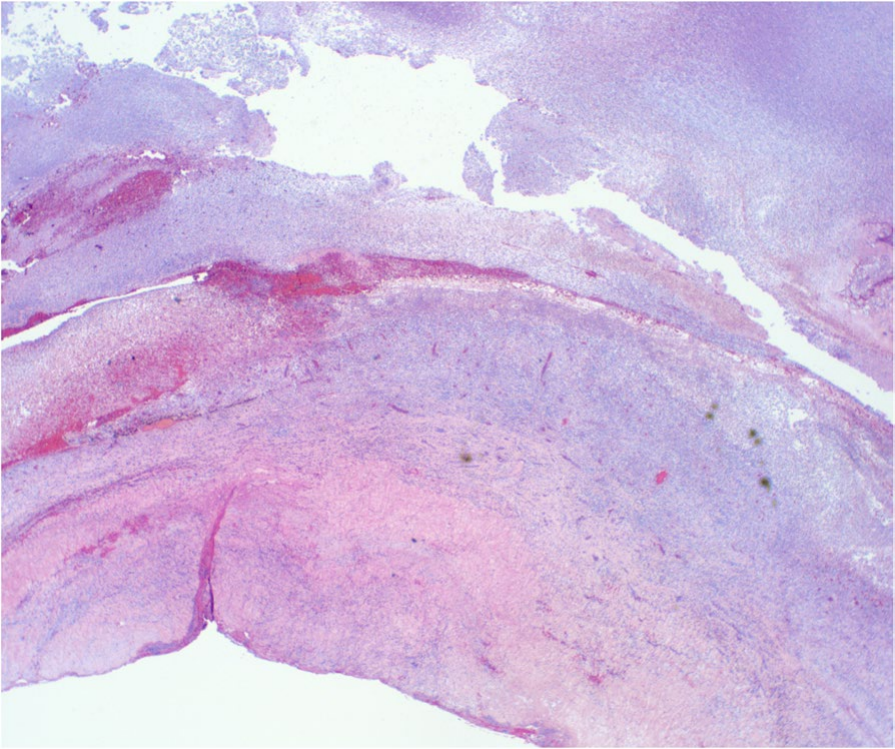
Shows a low power view showing a dense inflammatory infiltrate of the extremely thickened sclera with foci of hemorrhage

There is extensive destruction of intraocular tissues. Figure 2.

The corneal epithelium was absent and the endothelial numbers are decreased and the posterior stroma shows marked inflammatory cells. There are some keratic precipitates. There is hemorrhage in the anterior chamber and the angle appears narrow with anterior displacement of the ciliary body complex causing angle closure. The lens structures are disturbed with anterior capsular remnant visible but the bulk of the lens is missing and there is considerable inflammation near the posterior capsule.

**Fig. 3:**
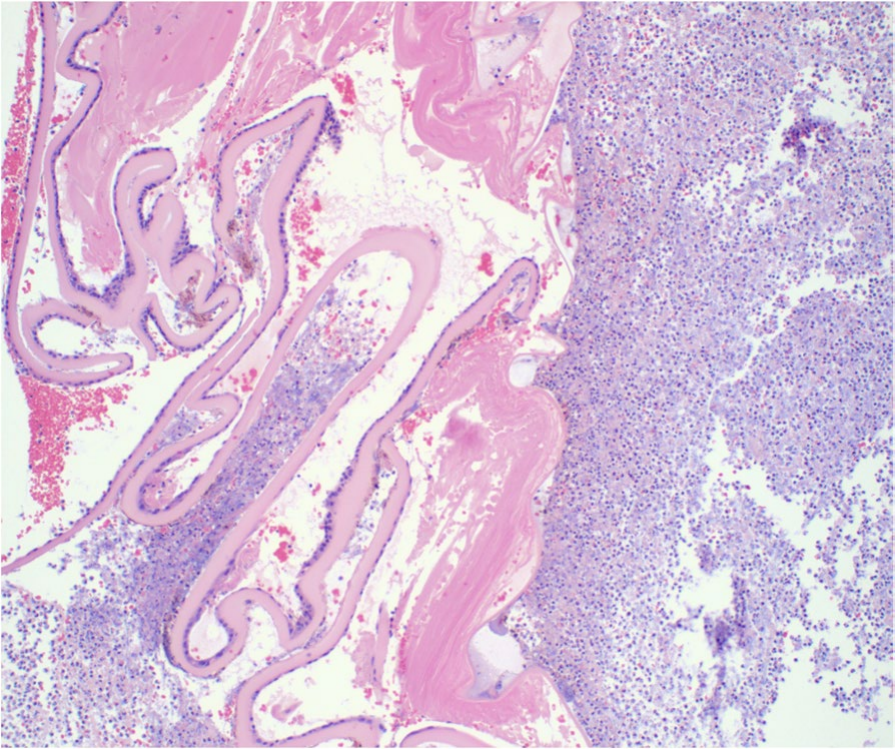
Shows a medium power view showing remnants of the anterior lens capsule with zonal inflammation posteriorly. There is very little lens material remaining within the segment shown and there is a dense inflammatory infiltrate posterior the lens capsule

The vitreous cavity is massively filled with inflammatory cells Fig. 3. There is marked retinal degeneration. The choroid has acute and chronic inflammatory cells. There were no microorganisms seen on the Gram, PAS, and GMS Stains.

## Discussion

MRSA orbital cellulitis is an aggressive disease and generally caused by external trauma or injury that seeds the gram-positive organism posterior to the orbital septum, although it can also be caused endogenously through systemic infection. Bilateral blindness from community-associated MRSA orbital cellulitis has also been seen in the literature. This was reported in an otherwise healthy incarcerated 44-year-old man and developed fever and chills two days after squeezing a pustule on his nares. There were no additional risk factors such as HIV, diabetes, steroid use, or hospitalization. By Day 11 he progressed to bilateral no-light-perception and cavernous sinus thrombosis. Our case report involves an episode of MRSA panophthalmitis that we believe disseminated endogenously secondary to the patient’s infected central line. Our patient also suffered from common risk factors that predisposed him to severe infection including poorly controlled diabetes, renal dysfunction on hemodialysis in addition to the infected central line.

A work up for EBE should include systemic evaluation including CBC, CMP, ESR/CRP, and blood cultures along with ophthalmic consultation. In cases of MRSA endogenous endophthalmitis, blood cultures have much greater diagnostic yield than vitreous cultures with sensitivity of 76% and 56%, respectively [3]. Although most literature supports a vitreous tap in the initial work-up and management of suspected EBE, only 12% of vitreous cultures grew MRSA. In contrast, in a study of 17 cases of MRSA EBE, 100% of patients had blood cultures positive for MRSA [7]. In cases of MRSA EBE secondary to infected catheters or central lines, the hardware was removed upon treatment initiation. It is unclear if this is beneficial, but it is recommended that these devices be removed in the setting of any MRSA bacteremia.

While there are very few reported cases of MRSA EBE, intravenous administration of vancomycin was the common choice of antibiotic for treatment. However, systemic vancomycin has an unpredictable penetration into the brain-retinal barrier and into the vitreous body. Studies of general endophthalmitis cases showed that vancomycin penetration was poor in the vitreous one and five hours after 1 IV gram dose with the highest serum concentration of 20% [8]. Whereas perhaps in cases of endogenous endophthalmitis with greater systemic infection and theoretically meningeal inflammation, IV vancomycin may have better penetration [8]. As such, IV antibiotics are a cornerstone of EBE, but they are not recommended in cases of exogenous or localized endophthalmitis.

Other IV antibiotics have been utilized with varying success in MRSA endogenous endophthalmitis including: daptomycin, ceftaroline, and linezolid [1]. Daptomycin has a very poor ocular penetration, estimated to be around 28%, but is favorable in that it is not metabolized by kidneys as many of these patients have kidney dysfunction [8]. Studies have shown very favorable intravitreal penetration at Linezolid as another potential antibiotic with fair ocular penetration; however, it is bacteriostatic and doesn't directly eliminate bacteria. Linezolid has been used in MRSA endogenous endophthalmitis with success but it is not recommended as a first line antibiotic due to side effects of bone marrow suppression and optic neuropathy. Of all antibiotic classes, fluoroquinolones have greatest penetration of the vitreous when given intravenously; however, there is a high resistance for this class of antibiotics among MRSA so they are not recommended as first line in MRSA endogenous endophthalmitis [8]. Ceftaroline has shown promising results in a case series of MRSA endogenous endophthalmitis with recent studies have found it to have better vitreous penetration and a better side effect profile than vancomycin, daptomycin, and linezolid [1].

The prognosis for MRSA EBE is usually poor, but it is difficult to prognosticate because the few publications on MRSA EBE involved are case series. Studies have shown retinal detachment as a common sequelae of MRSA EBE; the rate of retinal detachment has been estimated between 53 to 75% [1, 7]. Both studies however did not find a significant correlation between poor visual acuity (> 20/200) and presence of a retinal detachment.

One large retrospective study found that 47% of patients had a final visual acuity worse than 20/200. Furthermore this study found that poor visual acuity was not significantly affected by phakia status, diabetes mellitus, overall systemic illness, or development of retinal detachment, like the two studies above [7]. The significant difference between patients who recovered with a visual acuity less than 20/200 versus worse than 20/200 (*p* = 0.02) was time of admission to endophthalmitis diagnosis [7]. A literature review of case reports and case series of EBE, not specific to MRSA EBE, found that 44% resulted in total blindness while 25% required evisceration or enucleation in the end [3].

## Conclusions

In summary, we present a case of rapidly progressive MRSA associated orbital panophthalmitis likely endogenously spread from an infected permanent line central catheter. Risk factors predisposing patients to developing MRSA EBE include diabetes, renal dysfunction, immunocompromised state, and presence of central or venous lines. While systemic illness does predispose patients to the development of MRSA EBE, it does not correlate to worse visual outcomes. Although visual prognosis for MRSA EBE is poor, timely diagnosis and treatment with IV antibiotics and ophthalmic consultation aid in vision preservation. Intravenous antibiotics and removal of infected hardware remain the basis of management for MRSA EBE. Tap for vitreous sampling and injection of intravitreal antibiotics is recommended in the presence of vitreal or posterior involvement of the infection. Yield of vitreous sampling for culture has been shown to be less sensitive as blood cultures, and surgical intervention in the presence of retinal detachment has shown some benefit when done early in patient presentation.

## Data Availability

Glass slides are available upon request or photos thereof are also available.
